# *Vibrio cholerae* O1 Hybrid El Tor Strains, Asia and Africa

**DOI:** 10.3201/eid1406.080129

**Published:** 2008-06

**Authors:** Ashrafus Safa, Jinath Sultana, Phung Dac Cam, James C. Mwansa, Richard Y.C. Kong

**Affiliations:** * City University of Hong Kong, Hong Kong Special Administrative Region, People’s Republic of China; † National Institute of Hygiene and Epidemiology, Hanoi, Vietnam; ‡ University Teaching Hospital, Lusaka, Zambia

**Keywords:** Vibrio cholerae O1, classical, El Tor, hybrid El Tor, cholera toxin, ctxB, letter

**To the Editor:** *Vibrio cholerae* is a water-borne pathogen that causes a severe watery diarrhea disease known as cholera. On the basis of variable somatic O antigen composition, >200 serogroups of *V. cholerae* have been recognized. Classical and El Tor are 2 well-established biotypes within the *V. cholerae* O1 serogroup, and they can be distinguished by differences in their biochemical reactions or phenotypic traits (*1*). In addition to phenotypic traits, genetic markers have recently been used in the identification of the biotypes of *V. cholerae*. For example, the major toxin-coregulated pilus (TCP) gene, *tcpA*, of the TCP cluster possesses classical- and El Tor–specific alleles that encode identical functions but differ in their DNA sequence composition; however, the *rtxC* gene of the repeat in toxin (RTX) cluster is present in El Tor strains only and absent in classical strains (*2*,*3*). The cholera toxin, encoded by the *ctxA* and *ctxB* genes, is the principal toxin produced by *V. cholerae* O1 and O139 and is responsible for the disease cholera. Heterogeneity within the *ctxB* gene and protein was first reported in the early 1990s, and, on this basis, 3 *ctxB* genotypes of the *V. cholerae* O1 strains have been identified. Based on amino acid residue substitutions at positions 39, 46, and 68, all classical and US Gulf Coast El Tor strains have been categorized as genotype 1, the Australian El Tor strains as genotype 2, and the El Tor strains of the seventh pandemic and the Latin American epidemic as genotype 3. Genotyping of *ctxB* has indicated that the classical strains harbor a unique cholera toxin gene that is not in the El Tor strains except for the US Gulf Coast El Tor clone (*4*). The US Gulf Coast hybrid El Tor strains that harbor the classical cholera toxin have been associated with sporadic outbreaks in the United States (*5*) and, until recently, had not been reported anywhere else in the world. Then in 2004, hybrid El Tor strains that encode the classical cholera toxin were isolated from cholera patients in Matlab, Bangladesh (*6*), and in Beira, Mozambique (*7*). In 2006, Nair et al. reported that the current seventh pandemic prototype El Tor strains had been replaced by hybrid El Tor strains in Bangladesh (*8*). We now report how far the hybrid El Tor strains have spread in Asia and Africa.

We examined 41 clinical *V. cholerae* strains from Asia and Africa that were isolated from 1991 through 2004 (Table) and confirmed as serogroup O1 by O-antigen biosynthesis gene (*rfbO1*)-specific PCR. Biotyping was performed by using standard procedures, and all strains were confirmed as El Tor (Table). All strains were PCR-positive for the El Tor–specific 451-bp *tcpA* and 263-bp *rtxC* amplicons but negative for the classical-specific 620-bp *tcpA* amplicon. All 41 strains were PCR-positive for *ctxAB* (1,037 bp) and produced cholera toxin, as demonstrated by the VET-RPLA Toxin Detection Kit (Oxoid, Basingstoke, UK). Sequence comparison of the PCR-amplified *ctxB* gene (460 bp) of each strain with the reference strains (569B and N16961) showed that 30 strains harbored classical cholera toxin (with histidine at position 39, phenylalanine at position 46, and threonine at position 68), whereas the remaining 11 strains carried the El Tor cholera toxin gene (with tyrosine at position 39, phenylalanine at position 46, and isoleucine at position 68) (Table). The overall analysis showed that all test strains are El Tor biotype but that most harbor the classical cholera toxin gene.

**Table:** Phenotypic and genotypic traits of *Vibrio cholerae* O1 clinical strains isolated from Asia and Africa, 1991–2004*

Test strain origin (no. examined) or reference strain ID	Year(s) of isolation	*rfbO1*	Phenotypic tests				PCR amplicons			Cholera toxin type† (no. strains)
			CCA	Polymyxin B (50 U)	Voges-Proskauer		*tcpA* (El Tor)	*tcpA* (classical)	*rtxC*	
Japan (6)	1991–1997	+	+	Resistant	+		+	–	+	Classical (5), El Tor (1)
Hong Kong (18)	1998–2000	+	+	Resistant	+		+	–	+	Classical (11), El Tor (7)
Zambia (8)	1996–2004	+	+	Resistant	+		+	–	+	Classical (5), El Tor (3)
China (3)	1999	+	+	Resistant	+		+	–	+	Classical (3), El Tor (0)
Sri Lanka (1)	1998	+	+	Resistant	+		+	–	+	Classical (1), El Tor (0)
Vietnam (5)	1994–2002	+	+	Resistant	+		+	–	+	Classical (5), El Tor (0)
Classical 569B	1948	+	–	Sensitive	–		–	+	–	Classical
El Tor N16961	1971	+	+	Resistant	+		+	–	+	El Tor

*rfbO1, O-antigen biosynthesis genes; CCA, chicken cell agglutination; tcp, toxin-coregulated pilus; rtx, repeat in toxin. †Based on *ctxB* sequence. GenBank accession nos. for the *ctxB* sequences are EU156448–EU156488.

The major finding of this study is that El Tor strains that harbor the classical cholera toxin gene are not limited to the US Gulf Coast, Bangladesh, and Mozambique; they have spread to several other countries in Asia and Africa. Since 1817, 7 cholera pandemics have occurred around the world. Firm evidence indicates that the fifth and sixth cholera pandemics were caused by the classical biotype whereas the most extensive and ongoing seventh pandemic is caused by the El Tor biotype. Since the onset of El Tor dominance in 1961, the classical strains have been gradually replaced by the El Tor strains and are now believed to be extinct. However, reports from Bangladesh (*6*), Mozambique (*7*), and this study have provided sufficient evidence to indicate that the classical cholera toxin gene has reappeared but that for these cases its carrier has been El Tor. Although how the classical cholera toxin in El Tor strains would affect *V. cholerae* pathogenicity is unclear, cholera caused by the classical biotype is more severe, whereas the El Tor biotype is considered to be better able to survive in the environment (*1*,*9*). Given that cholera toxin is directly responsible for the major clinical sign of the disease, such a genetic change could result in substantial alteration in the clinical manifestation of cholera. Additionally, this subtle genetic change may also influence the effectiveness of current cholera vaccines, which could stimulate both antitoxic and antibacterial immunity.
